# Supplementary material for: The use of restricted mean time lost under competing risks data
Source: BMC Med Res Methodol. 2020 Jul 25;20:197. doi: 10.1186/s12874-020-01040-9 (PMC7382086; doi:10.1186/s12874-020-01040-9)
Supplement: Supplementary file 1 — Additional file 1. [file 12874_2020_1040_MOESM1_ESM.pdf]

Supplemental Captions

Appendix A: Theory of variance techniques (ANOVA) as a supplementary analysis to evaluate the type I error and power.

Table A1: The parameter settings of the two CIFs for the simulations.

Table A2: Comparison of power under a fixed sample size.

Appendix A: Theory of variance techniques (ANOVA) as an assistant analysis to evaluate the type I error and power.

Following Klein et al. (2007) and Logan et al. (2008), we applied analysis of variance (ANOVA) techniques to evaluate both the type I error and the power. The response variable Y for evaluating the type I error is defined as the percent rejection rate minus the nominal 0.05 level. Therefore, good test performance is indicated by absolute small, close-to-zero estimates of the expectation $E(Y)$ in the ANOVA. To evaluate power, the outcome variable Y is defined as the percent rejection rate, and good performance is indicated by large estimated values of $E(Y)$. Here, we considered four different factors: *test* for the testing methods; $n_1\_n_2$ for the sample size of each group; *sit* for the simulated situation; and *cen* for the censoring rate. We illustrate the performance of the tests by fitting the following four models of $E(Y)$:

Model 1: $E(Y) = test \times n1\_n2 + cen + sit$

Model 2: $E(Y) = test \times cen + n1\_n2 + sit$

Model 3: $E(Y) = test \times sit + n1\_n2 + cen$

Model 4: $E(Y) = test + n1\_n2 + cen + sit$

REFERENCES

Klein JP, Logan B, Harhoff M, Andersen PK. Analyzing survival curves at a fixed point in time. Statistics in Medicine. 2007; 26, 4505-4519.

Logan BR, Klein JP, Zhang MJ. Comparing treatments in the presence of crossing survival curves: an application to bone marrow transplantation. Biometrics. 2008; 64(3), 733-740.

Table A1:

The parameter settings of the two CIFs for the power simulations.

Situation	Control group	Experimental group
A	*exp*(1)	*exp*(1)
B	*exp*(1.649)	*exp*(1)
C	*w*(2,2)	*w*(0.5,2)*I*(*t*≤0.5)+*w*(2,2)*I*(*t*>0.5)
D	*w*(1,2)*I*(*t*≤2)+*w*(2,2)*I*(t>2)	*w*(4,2)*I*(*t*≤2)+*w*(2,2)*I*(*t*>2)
E	*w*(0.8,2)	*w*(0.8,2)*I*(*t*≤2)+*w*(0.3,2)*I*(*t*>2)
F	*w*(3,2) *I*(*t*≤2.5)+*w*(2,2)*I*(*t*>2.5)	*w*(0.6,2) *I*(*t*≤2.5)+*w*(2,2)*I*(*t*>2.5)

exp(*parameter*): exponential distribution; *w*(*parameter*, *scale*): Weibull distribution.

Table A2:

Comparison of power under a fixed sample size.

*n**	Power		
	Gray	Diff	sDiff
116	0.859	0.878	0.790
220	0.390	0.893	0.886
152	0.935	0.961	0.898
294	0.497	0.949	0.965
168	0.830	0.879	0.776
322	0.269	0.872	0.841
212	0.926	0.949	0.889
408	0.375	0.930	0.940

*: sample size of sDiff test calculated in Table 4.
